# Consumption of Citric Acid by Bees Promotes the Gland Development and Enhances Royal Jelly Quality

**DOI:** 10.3390/life14030340

**Published:** 2024-03-06

**Authors:** Xue Wang, Quanzhi Ji, Xing Zheng, Jun Zhang, Rongshen Wang, Xinyu Wang, Wenjun Peng, Jun Guo, Yazhou Zhao

**Affiliations:** 1 State Key Laboratory of Resource Insects, Institute of Apicultural Research, Chinese Academy of Agricultural Sciences, Beijing 100093, China; 82101212175@caas.cn (X.W.); 18336369851@163.com (Q.J.); zhengxing@caas.cn (X.Z.); pengwenjun@vip.sina.com (W.P.); 2 College of Bee Science, Fujian Agriculture and Forestry University, Fuzhou 350000, China; 3 Faculty of Life Science and Technology, Kunming University of Science and Technology, Kunming 650500, China; 4 Shijiazhuang Animal Disease Prevention and Control Center, Shijiazhuang 050026, China

**Keywords:** honeybees, citric acid, glands development, royal jelly quality

## Abstract

The glands of bees are responsible for generating and secreting various biologically active substances that significantly impact bee physiological health and adaptability. This study aimed to investigate the effects of adding citric acid (CA) to bee feed on gland development and royal jelly quality. By formulating feed with varying proportions of CA, evaluation was undertaken of pollen feeding by honeybees under laboratory conditions, along with the impact of CA on the development of major glands, to determine suitable addition proportions. Further optimization of the CA proportion involved feeding colonies and evaluating royal jelly production and quality. The results indicated that feed containing 0.75% CA significantly extended the lifespan of bees and increased their pollen consumption. Gland development in bees showed a positive correlation with CA addition within the range of 0.25% to 0.75%, especially at 0.50% and 0.75%, which notably accelerated the development of mandibular, hypopharyngeal, and cephalic salivary glands, with active proliferation and differentiation of glandular cells and maintenance of normal gland size and morphology. CA added to feed stimulated vigorous secretion of wax glands in worker bees, resulting in prolific wax construction. Colonies consuming feed containing 0.50% CA produced royal jelly with significantly reduced moisture and total sugar content and increased levels of 10-HDA, total phenolic acids, total proteins, and acidity. These findings demonstrate that CA consumption significantly prolongs bee lifespan, increases consumption, promotes gland development, and enhances royal jelly quality. This research provides theoretical guidance for beekeeping practices and feed development, contributing to the sustainable advancement of apiculture.

## 1. Introduction

Bees play a crucial role as vital pollinators of crops and primary producers of bee-related products, significantly impacting agricultural production and human health [1,2]. The glands of bees, including the mandibular glands (MGs), hypopharyngeal glands (HGs), salivary glands, post-cerebral glands, and wax glands, are responsible for secreting or storing various biologically active substances, such as saliva, honey, and wax, supporting the function of bees as pollinators [3]. Among these, the MGs are situated beneath the mandibular plate in the head of bees and include secretory tissue and storage sacs [3]. They are mainly involved in the synthesis and secretion of compounds such as 10-hydroxy-2E-decenoic acid (10-HDA) [4] and alarm pheromones such as 2-heptanone [5]. The HGs are a pair of external secretion glands in the head of bees, each consisting of elongated, grape-like secretory acini [6]. HGs are responsible for secreting royal jelly, with the most robust development observed in 6- to 12-day-old worker bees [7]. Subsequently, following the development of wax glands, the HGs begin to atrophy and regress [4]. The salivary glands comprise the cephalic salivary glands (CSGs) and the thoracic salivary glands (TSGs) [8]. Each CSG is composed of two flattened pear-shaped bodies. The CSGs of worker bees contained numerous grape-like acini and several branched ducts [9]. The CSGs are usually intertwined with the HGs and responsible for saliva secretion, aiding in food digestion in bees [10]. TSG secretions are primarily utilized in bee cleaning and hygiene behaviors [10]. Moreover, wax glands are located in the abdominal segments of worker bees and are composed of numerous glandular lobes responsible for wax secretion, which is essential for nest construction and sealing [11].

The development and functionality of bee glands are regulated by various factors, among which dietary additives, such as organic acids, are considered significant influencers [12,13]. Organic acids such as citric acid (CA) actively participate in the metabolism of sugars, fats, and proteins within organisms and are extensively applied in the food and feed industries [14]. Apart from their roles in flavor enhancement and acidification, organic acids can modulate food intake, metabolism, and gene expression [15,16,17]. These compounds stimulate the proliferation and differentiation of glandular cells, thereby promoting gland development and enhancing functionality [18,19,20]. CA, an intermediate in the tricarboxylic acid cycle, is associated with fatty acid synthesis [21]. Studies have shown that the addition of organic acids (lactic acid and acetic acid) to feed significantly increases the acinar surface area of worker bee HGs, fostering gland growth and development and consequently leading to increased secretion of royal jelly [22]. Zhang et al. discovered that feeding CA enhances protein concentrations in the MGs of worker bees, stimulates the beta-oxidation process of fatty acids, and improves bee energy supply, ultimately increasing the quality of the secreted royal jelly [23].

Previous research commonly revealed that organic acids facilitate bee gland development and increase royal jelly production [22,24]. However, the exact impact model and underlying mechanisms, including the dose–response relationship and evaluation of royal jelly quality, remain ambiguous. This study aimed to explore the influence of incorporating different ratios of CA into hive feed on the development of key bee glands and the quality of the resulting royal jelly. This investigation sought to unveil the regulatory mechanisms governing bee gland development and offer strategies for enhancing the quality of royal jelly products.

## 2. Materials and Methods

### 2.1. Experimental Subjects

The experimental subjects consisted of Western honeybees (*Apis mellifera* L.) from the experimental apiary of the Chinese Academy of Agricultural Sciences, Institute of Apicultural Research. Healthy and active honeybees newly emerged from the same colony were selected as experimental subjects to ensure that they were in a similar physiological state.

### 2.2. Reagents and Equipment

White Vaseline (Xilong Scientific, Shantou, China), CA (Macklin, Shanghai, China), sucrose (Xilong Scientific, Shantou, China), and rapeseed pollen were obtained from Jin’s Beekeeping Cooperative in Yifeng County, Yichun City, Jiangxi Province. Disposable 2 mL sterile syringes (Zhiyu, Shanghai, China), a constant-temperature and -humidity incubator (Shanghai Yiheng Scientific Instruments Co., Ltd., Shanghai, China), an electronic balance (Shimadzu UW620H, Kyoto, Japan), fine forceps (FST by DUMONT 11252-20, DUMONT 0208-5-PO, Montignez, Switzerland), wax dishes (molten beeswax poured into Petri dishes after filtration), pathological-grade microscope slides (Shitai, Yancheng, China), microscope cover slides (Shitai, Yancheng, China), and a stereomicroscope (Leica EZ4 W, Wetzlar, Germany) were used.

### 2.3. Experimental Procedure

#### 2.3.1. Feed Preparation

The feed was prepared with varying proportions of CA. In the initial screening experiment, CA was added at proportions of 0% (control group), 1.00%, 2.00%, 3.00%, 4.00%, and 5.00%. Based on the results of the initial screening, the CA addition proportion was adjusted to 0% (control group), 0.25%, 0.50%, and 0.75%.

For feed preparation, 50% (*w*/*v*) sucrose solution was prepared first, followed by dissolving CA with different added ratios in sucrose solution, then mixing rapeseed pollen and citric acid-containing sucrose solution according to a mass ratio of 1:0.7, kneading to a non-sticky paste consistency, and making pollen cakes.

#### 2.3.2. Colony Rearing and Sampling Time

**Figure 1:**
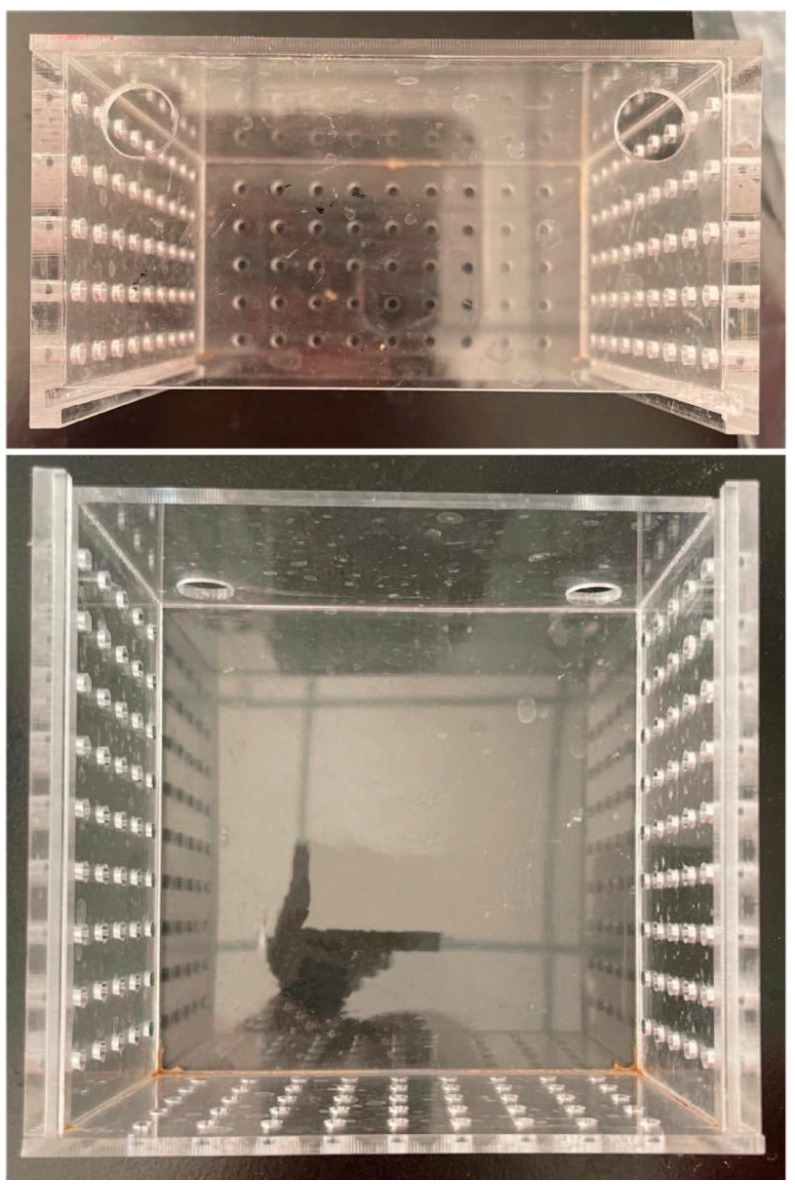
Feeding cage.

The rearing cages were constructed using transparent acrylic panels, as shown in Figure 1. The bottom and sides of the cages were equipped with small ventilation holes to ensure adequate air supply. Two 2 mL syringes were inserted at the top of each cage for feeding with a 50% (*w*/*v*) sucrose solution or water. Each cage contained a 35 mm petri dish to hold the feed.

Before the commencement of the experiment, two mature, sealed brood frames were retrieved from the bee colony and placed in a constant temperature and humidity incubator at 34 °C and 54% relative humidity (RH). Thirty newly emerged bees, less than 24 h old, were randomly selected and placed in each rearing cage. Each experimental group consisted of four rearing cages, all maintained at 32 °C and 75% RH. On a daily basis, each rearing cage received 1.5 mL of sucrose solution, 1.5 mL of water, and 1.0 g of feed, and the consumption was recorded, as was the number of deceased bees, which were removed from the feeding cage.

To determine the evaporation loss of sucrose, water, and feed under identical experimental conditions, four rearing cages without bees but containing sucrose solution, water, and feed were placed in the same constant-temperature and -humidity incubator [25]. Daily replacement and weighing were conducted to ascertain the evaporative loss. The daily food consumption per bee was calculated based on the total consumption and the number of surviving bees. A total of four bees from each group of rearing cages were randomly sampled on the 6th, 9th, 12th, 15th, and 18th days and then immediately frozen in liquid nitrogen before being stored at −80 °C for future analysis.

#### 2.3.3. Gland Isolation and Measurement

Fresh bee samples were secured onto wax dishes by tilting them with two entomological needles through the thorax and fixing them in place. Using forceps to stabilize the bee’s head, a small incision was made around the periphery of the compound eye, cutting through the chitin. After the edge of the maxillary chitin was grasped, the chitin covering the brain was gently lifted upward along the incision, exposing the brain tissue. The chitin covering the brain was carefully lifted, revealing the brain tissue, and the glands were gently separated using forceps [26,27].

When isolating, the chitin was removed from the bee’s brain, the extra chitin shell attached to the beak was removed, the head was clamped, and the part outside the MGs was clamped with tweezers and gently pulled it out. When isolating the HGs, the compound eye was first removed so as to prevent the rupture of the eye from contaminating the HGs and other tissues; the HGs were held with tweezers and the whole HGs were pulled out, and the impurities adhering to the HGs were then removed. Clean HG tissue could then be obtained. The CSGs are located in a string on the left and right at the bottom of the compound eye on the dorsal side of the head of the bee, and are usually entangled with the HGs. The shape of the CSGs is obviously different from that of the HGs. When dissecting the HGs, the CSGs only need to be pulled out of them.

The isolated glands were placed on microscope slides and observed and photographed under a Leica EZ4 W microscope at 12.5× magnification to determine the morphology of the HGs and at 20× magnification to determine the morphology of the MGs and CSGs. The color and fullness of the HG are essential indicators of its development; HGs are generally characterized by a milky color and being well rounded [28,29]. HG microscopic images were imported into Photoshop 2020 to measure the length and width of the HGs’ acini. Four bees per group were measured, with ten acini measured per bee, to calculate the surface area: $Acinal\;surface\;area=\pi \frac{ab}{2}$ (where *a* is the length, *b* is the width, and *π* = 3.14) [22].

#### 2.3.4. Colony Feeding and Royal Jelly Production

The colony feeding experiment was conducted in mid-July, during a period when external pollen sources were scarce. Three bee colonies were randomly selected from the apiary to ensure similar queen quality and colony strength [30]. These colonies were separately fed with CA addition ratios of 0% (control group), 0.50%, and 0.75%. The pollen cake was covered with a thin film to prevent water evaporation and placed on the comb frame. Bees generally fed along and under the edges of the pollen cake. Feeding was conducted twice a week, approximately 250 g each time, for a duration of 14 days. Three days after the feeding experiment was concluded, samples of royal jelly were collected and stored at −20 °C for further analysis [31].

#### 2.3.5. Determination of the Major Components of Royal Jelly

(1) Moisture Content

The moisture content in the royal jelly samples was determined using a vacuum drying oven (JWINS DZF-6020S, Shanghai, China). The samples were dried at 75 °C and −0.095 MPa to −0.10 MPa pressure for 4 h. After drying, the samples were cooled for 30 min. The drying process was repeated until the difference in mass between two consecutive dryings did not exceed 2 mg. The formula $X1=\frac{m1-m2}{m1-m3}\times 100$ was used to calculate the water content (where *X*1 = the water content in the royal jelly; *m*1 = the mass of the weighing bottle and sample; *m*2 = the mass of the weighing bottle and sample after drying to a constant weight; and *m*3 = the mass of the weighing bottle) [32].

(2) 10-HDA Content

The procedure was as follows: Accurately pipette 0.5, 1, 2, 3, 4, 5 mL of 10-HDA standard solution (1 mg/mL) into a 10 mL volumetric flask, precisely add 2 mL of internal standard solution (0.65 mg/mL 4-(carbomethoxy)phenol), dilute with anhydrous ethanol to the mark, and shake well. Separately take 2 μL of this solution. Using the peak area ratio calculation, it should be linear and the correction factor *F* should be calculated. After thawing the sample to room temperature, stir well with a glass rod, take about 0.5 g, and place it in a weighed 50 mL volumetric flask. Add 1 mL of 0.03 mol/L hydrochloric acid and 2 mL of distilled water, vortex and mix well to dissolve the sample. Add 30 mL of anhydrous ethanol, gently shake while adding, and then precisely add 10 mL of internal standard solution (0.65 mg/mL of 4-(carbomethoxy)phenol). Dilute with anhydrous ethanol to the mark, shake well, and immediately sonicate in an ultrasonic bath for 15 min before removing. Centrifuge at 3000 r/min for 10 min, then aspirate 4 μL solution into the chromatograph for measurement. The conditions for high-performance liquid chromatography (SHINADZU LC-10A, Japan) are as follows: mobile phase (CH_3_OH + 0.03 mol/L HCl + H_2_O) = 55 + 10 + 35, wavelength 210 nm, column temperature 35 °C, mobile phase flow rate 1 mL/min. The formula $X2=F\times \frac{Ai}{As}\times \frac{mi}{ms}\times 100$ was used to calculate the 10-HDA content (where *X2* = the 10-HDA content in the royal jelly, *F* is the correction factor, *Ai* is the peak area of the tested component in the sample, *As* is the peak area of the internal standard in the sample, *ms* is the mass of the internal standard, *mi* is the mass of the sample) [32].

(3) Total Protein Content

The total protein content was determined using the Kjeldahl method. A measured amount of each royal jelly sample was placed in a Kjeldahl flask for digestion. The digestion solution was distilled using a semimicro distillation apparatus. Subsequently, a 0.01 mol/L hydrochloric acid standard solution was titrated into the absorption solution. A change in color from bluish-green to grayish-purple indicated the endpoint. The total protein content (*X*3) was calculated using the formula $X3=\frac{(V1-V0)\times C1\times 0.014}{m4\times \frac{5}{100}}\times 6.25\times 100$, where *X*3 represents the total protein content in royal jelly, *v*1 is the volume of 0.01 mol/L hydrochloric acid standard solution consumed during titration of the sample, *v*0 is the volume of 0.01 mol/L hydrochloric acid standard solution consumed during the blank titration, *c*1 is the concentration of the hydrochloric acid standard solution, and *m*4 is the mass of the sample [32].

(4) Water-Soluble Protein Content

A 2 g royal jelly sample was diluted with distilled water to a volume of 10 mL. After complete dissolution, the solution was centrifuged at 12,000 rpm and 4 °C for 10 min. The supernatant was collected in a centrifuge tube for analysis. A Bradford protein quantitative assay kit (Beyotime) was used for analysis. Protein standard: Take 1.2 mL of protein standard preparation solution and add it to a tube of protein standard (30 mg BSA). Dissolve it thoroughly and prepare a 25 mg/mL protein standard solution. Take an appropriate amount of 25 mg/mL protein standard and dilute to a final concentration of 0.5 mg/mL. According to the sample, prepare an appropriate amount of BCA working solution by adding 1 volume of BCA reagent B (50:1) to 50 volumes of BCA reagent A, and mix thoroughly. Divide standard products into 0, 1, 2, 4, 8, 12, 16, and 20 μL. Add it to the standard well of the 96-well plate and add standard diluent to make up to 20 μL, equivalent to standard concentrations of 0, 0.025, 0.05, 0.1, 0.2, 0.3, 0.4, and 0.5 mg/mL, respectively. Dilute each sample by 30, 40, and 50 times, and add it to the sample well of a 96-well plate. Add 20 μL to each hole. Place BCA working solution at 37 °C for 20–30 min. Measure with an enzyme-linked immunosorbent assay (ELISA) reader and calculate the protein concentration of the sample.

(5) Total Sugar Content

A 4 g sample of royal jelly was placed into a 100 mL volumetric flask and dissolved in 50 mL of distilled water. After dissolution, 5 mL each of zinc acetate solution and potassium ferrocyanide solution was added. The solution was adjusted to a predetermined volume with distilled water. After standing for 30 min, the solution was filtered through dry filter paper, after which the initial filtrate was discarded. Fifty milliliters of the filtrate was added to a 100 mL volumetric flask, to which 10 mL of hydrochloric acid (*c* = 6 mol/L) was added. The mixture was shaken and placed in a water bath maintained at a temperature range of 68 °C to 70 °C for 10 min for hydrolysis. After cooling to room temperature, 2 drops of methyl red indicator were added, and the solution was titrated with sodium hydroxide solution (ρ = 200 g/L) until the solution turned yellow. The solution was subsequently diluted with distilled water, shaken well, and used as the sample solution.

Specifically, 5 mL each of alkaline potassium tartrate-copper and iodide were added to a 150 mL conical flask, mixed with 10 mL of distilled water, and heated to boiling within 2 min. The sample solution was added dropwise at a fast rate initially and then slowly while maintaining a boiling state. When the solution color lightened, it was titrated at a rate of one drop every 2 s until the blue color just disappeared as the endpoint. The volume of sample solution consumed was recorded. The total sugar content was calculated as $X4=\frac{T}{m5\times \frac{v2}{100}\times \frac{1}{2}\times 1000}\times 100$, where *X*4 represents the total sugar content in royal jelly, *T* is the volume of alkaline potassium tartrate-copper solution required for titration (10 mL of alkaline potassium tartrate-copper solution equivalent to the mass of glucose), *m*5 is the mass of the sample, and *v*2 is the volume of sample solution consumed during titration [33].

(6) Acidity

One gram of each royal jelly sample was weighed into a 100 mL beaker, and 75 mL of distilled water was added. The solution was titrated with standard sodium hydroxide solution (*c* = 0.1 mol/L) until it reached a pH of 8.3 at the endpoint. The acidity (*X*5) was calculated using the formula X5=v3×c2×100, where *X*5 represents the acidity of the royal jelly, *v*3 is the volume of standard sodium hydroxide solution consumed during titration, and *c*2 is the concentration of the standard sodium hydroxide solution [34].

(7) Total Phenolic Acid Content

Standard solutions of gallic acid were prepared at concentrations of 0, 0.1, 0.2, 0.4, 0.6, 0.8, and 1.0 mL (100 μg/mL). To each solution, 1 mL of Folin–Ciocalteu reagent was added, and the solution was mixed thoroughly. After 3 min, 1 mol/L sodium carbonate solution was added, and the solution was diluted to 10 mL with distilled water. The mixture was allowed to stand in the dark for 1 h, after which the absorbance was measured at 760 nm. A standard curve was constructed using gallic acid concentrations (x) ranging from 0 to 100 μg/mL and their respective absorbance values (y), with the equation y = 0.0047x + 0.036 and a correlation coefficient R2 = 0.9939.

Five grams of each royal jelly sample was weighed in a 50 mL volumetric flask and dissolved in deionized water to a final volume of 50 mL. The mixture was sonicated for 10 min and centrifuged at 15,000 rpm for 10 min, after which the supernatant was diluted to 20 mg/mL. One milliliter of the 20 mg/mL royal jelly solution was added to 1 mL of Folin–Ciocalteu reagent, which was mixed well, and after 3 min, 1 mol/L sodium carbonate solution was added. The mixture was diluted to 10 mL with distilled water and left to stand in the dark for 1 h. The absorbance was measured at 760 nm, and the total phenolic acid content was calculated based on the absorbance values [35].

### 2.4. Data Analysis

Statistical analysis of the experimental results was performed using Microsoft Excel 16.17. Multiple sample comparisons and correlation analyses were conducted using SPSS 27.0 and the single factor ANOVA method. *p* < 0.05 indicates statistical significance. Graphs, including bar charts, line plots, and other visualizations, were created using GraphPad Prism 9.

## 3. Results

### 3.1. Survival Curve Analysis

**Figure 2:**
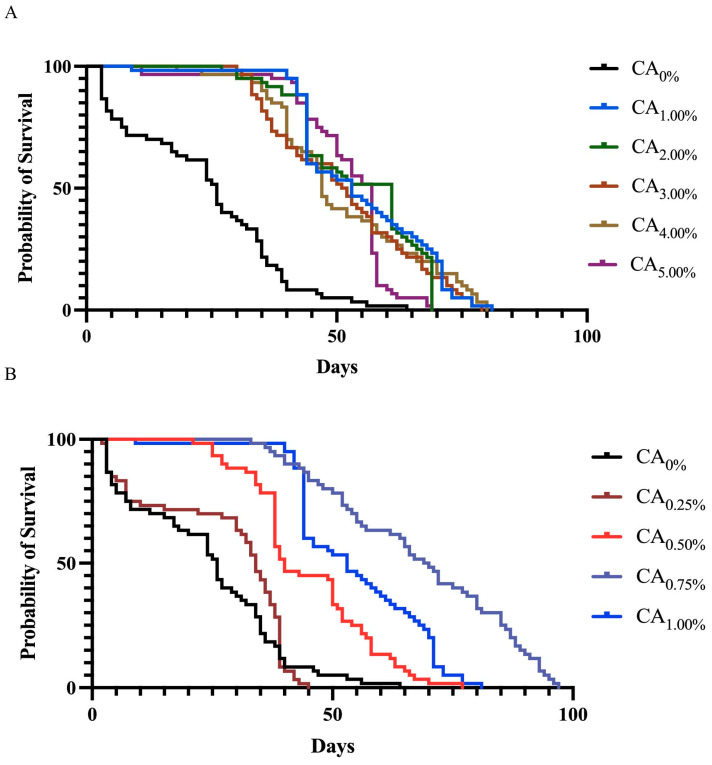
Effect of the proportion of CA added to the diet on the survival rate of honeybees. (**A**) Survival of treatment groups at 0%, 1.00%, 2.00%, 3.00%, 4.00%, and 5.00% CA addition to the diets. (**B**) Survival of treatment groups at 0%, 0.25%, 0.50%, 0.75%, and 1.00% CA addition to the diets.

Laboratory feeding experiments revealed no significant difference in survival rates among the treatment groups treated with CA at concentrations of 1.00%, 2.00%, 3.00%, 4.00%, or 5.00% (*p* = 0.169). However, all the treatment groups exhibited significantly greater survival rates than did the control group (CA_0%_) (*p* < 0.05) (Figure 2A). The average lifespan of the bees in the treatment groups ranged from 53.32% to 56.23%. Notably, the CA_1.00%_ group exhibited a 56.23% increase in bee lifespan. The outcomes revealed significant differences in survival rates between the two treatment groups (*p* < 0.05). Specifically, the CA_0.75%_ group exhibited significantly greater survival rates than the other groups, in which the average lifespan was extended by 64.73%.

### 3.2. The Impact of Adding Citric Acid to Bee Feed on Pollen Consumption

**Figure 3:**
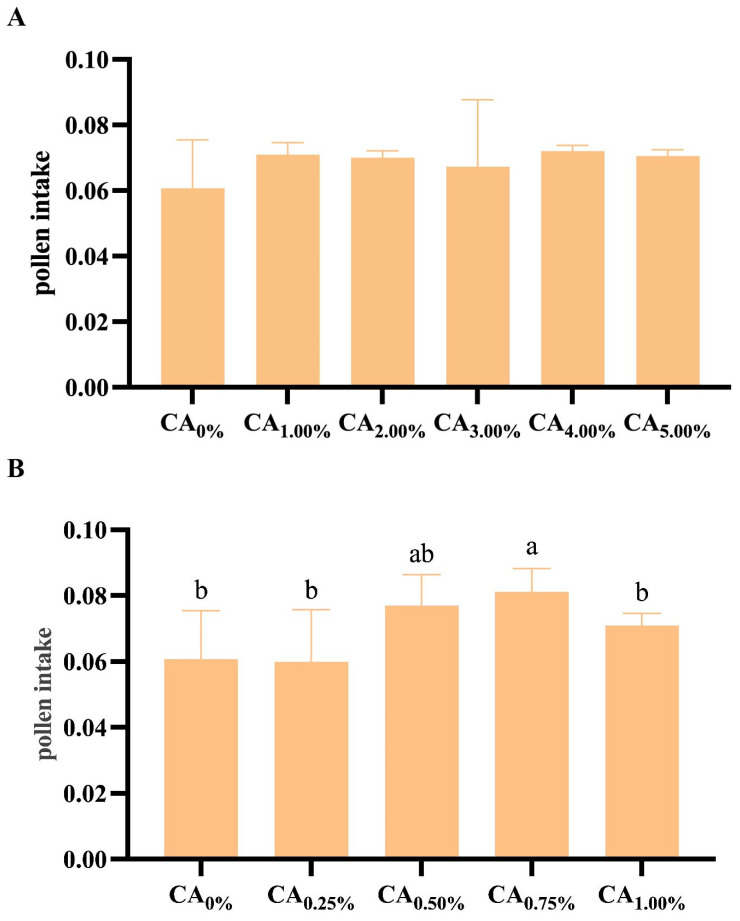
Effect of CA addition to the forage diet on pollen intake of honeybees. (**A**) Pollen intake by bees in each treatment group at 0%, 1.00%, 2.00%, 3.00%, 4.00%, and 5.00% CA addition to the diets. (**B**) Pollen intake by bees in each treatment group at 0%, 0.25%, 0.50%, 0.75% and 1.00% CA addition to the diets. Note: Different lowercase letters indicate significant differences (*p* < 0.05).

When CA was included at proportions of 0%, 1.00%, 2.00%, 3.00%, 4.00%, and 5.00%, no significant differences in pollen intake were observed across the treatment groups (*p* = 0.655) (Figure 3A). However, upon further refining the CA proportions to 0%, 0.25%, 0.50%, 0.75%, and 1.00%, the total amount of pollen consumed by each bee showed a trend of first increasing and then decreasing as the proportion of citric acid added increased (Figure 3B). Notably, at a CA proportion of 0.75%, the bees exhibited the highest pollen intake, significantly surpassing that of all the other treatment groups except for the CA_0.50%_ group (*p* < 0.05).

### 3.3. Effect of Citric Acid Addition to Feed on Bee Gland Development

#### 3.3.1. Mandibular Glands

**Figure 4:**
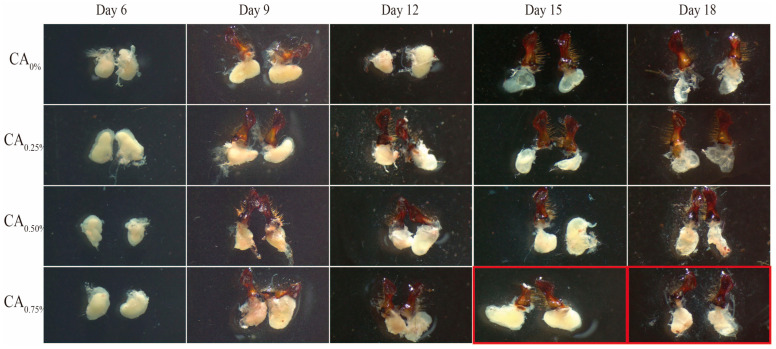
Effect of the proportion of CA added to the honeybee diet on the development of MGs. Note: The red box indicates significant differences in glandular development status.

The filling and morphology of cytoplasm are the main indicators used for evaluating the developmental status of MGs [36,37]. The experimental results showed that when the addition ratio was 0.75%, the vigorous period of MG development was significantly prolonged, the cytoplasm was filled and the morphology was good, indicating that the addition of CA in the diet had a significant impact on the development of bee MGs. As the proportion of CA added increased, it could significantly promote the development of the bee’s MGs (Figure 4).

#### 3.3.2. Hypopharyngeal Glands

**Figure 5:**
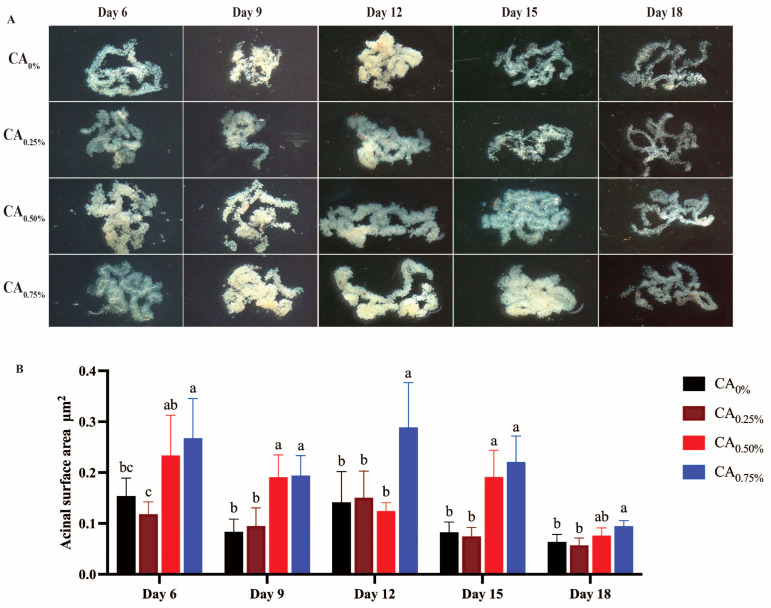
Effect of the proportion of honey bee diet supplemented with CA on the development of HGs. (**A**) HGs. (**B**) Acinar surface area. Note: Different lowercase letters indicate significant differences (*p* < 0.05).

As depicted in Figure 5, at 6 days old, the HGs of the CA_0.50%_ and CA_0.75%_ groups exhibited a milky color with fullness in the glands. Notably, the acinar surface area of the CA_0.75%_ group (0.284275225 ± 0.103194129) was significantly greater than that of the CA_0%_, CA_0.25%_, and CA_0.75%_ groups (*p* < 0.05). By 9 days old, the HGs gradually reached their developmental peak, with the CA_0.25%_ group showing a few glands with a water-white color and the majority exhibiting a milky color; moreover, the acinar surface areas of HGs in both the CA_0.50%_ group (0.19296433 ± 0.061531319) and the CA_0.75%_ group (0.199166291 ± 0.047600569) were significantly larger than those in the CA_0%_ group (0.08167719 ± 0.031423392) and the CA_0.25%_ group (0.095922511 ± 0.04620551) (*p* < 0.05). By 12 days old, the HGs of worker bees reached maturity, with full acini, a milky color, and rich and evenly distributed cytoplasmic contents. In particular, the acinar surface area of the CA_0.75%_ group was significantly greater than that of the other three groups (*p* < 0.05). At 15 and 18 days old, the HGs began to atrophy, and their color gradually shifted to water-white. However, the acinar surface area of the CA_0.50%_ and CA_0.75%_ groups remained larger than that of the CA_0%_ and CA_0.25%_ groups.

#### 3.3.3. Cephalic Salivary Glands

**Figure 6:**
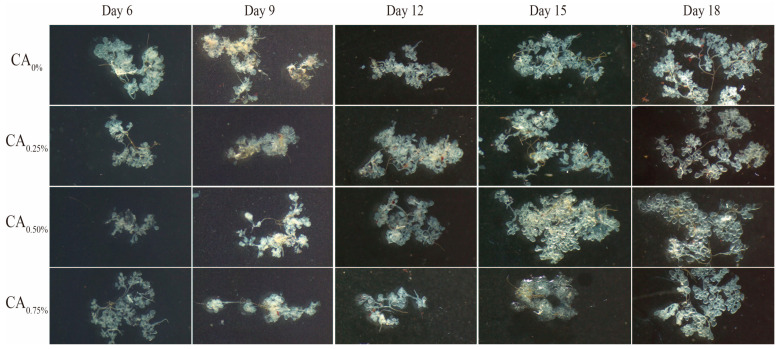
Effect of the proportion of CA added to the diet on the development of the CSGs.

As depicted in Figure 6, at 6 days old, the development of the worker bees HGs was incomplete. In the CA_0%_ group, individual acini appeared to be nearly circular or elliptical, with multiple acini stacking and aligning with each other. In the CA_0.25%_ and CA_0.50%_ groups, there was separation among the acini, with fewer connected duct branches. Notably, in the CA_0.75%_ group, there was a significant increase in the number of interconnected duct branches among the acini. By 9 to 15 days old, the CSGs of worker bees matured, with most acini resembling inverted pear shapes, expanding overall, exhibiting smooth surfaces, and lacking significant depressions. The volume of the acini noticeably increased during this period. At 18 days of age, individual acini exhibited fully inverted pear shapes with larger volumes, and the number of duct branches among the acini of the CA_0.75%_ group was notably greater than that in the other three treatment groups.

### 3.4. Effect of Citric Acid Addition to Feed on Royal Jelly Quality

Upon feeding experimental bee colonies feed supplemented with CA, we evaluated royal jelly production and its main components. As shown in Table 1, the results indicated that the moisture content of the royal jelly produced by the CA_0%_, CA_0.50%_, and CA_0.75%_ experimental groups ranged between 63.30% and 67.00%. Notably, the moisture content in the royal jelly samples of the CA_0.50%_ and CA_0.75%_ groups was significantly lower than that of the control group (CA_0%_) (*p* < 0.05), signifying a significant reduction in the moisture content of royal jelly when feeding citric acid to bee colonies. Moreover, the bee colony experiment revealed the influence of citric acid feeding on the 10-HDA content in royal jelly. The 10-HDA content in the CA_0%_ and CA_0.75%_ groups was lower than that in the CA_0.50%_ group, while that in the CA_0.50%_ group was significantly greater than that in the CA_0%_ group (*p* < 0.05). The total protein content in the royal jelly samples from each experimental group ranged between 12.0% and 13.3%. Interestingly, the total protein content was greater in the CA_0.75%_ group than in the CA_0%_ and CA_0.50%_ groups. The water-soluble protein content in the royal jelly samples from the experimental groups ranged from 8.81 g/100 g to 10.32 g/100 g, with no significant differences observed between the groups (*p* > 0.05). Additionally, the total sugar content in the royal jelly samples across the experimental groups ranged from 9.70% to 10.60%. Notably, the total sugar content in the CA_0.50%_ group was significantly lower than that in the CA_0%_ and CA_0.75%_ groups (*p* < 0.05), suggesting that the addition of 0.50% CA to feed significantly decreased the total sugar content in the royal jelly produced by bee colonies. The acidity of the royal jelly in the experimental groups ranged from 31.4 mL/100 g to 34.8 mL/100 g. The royal jelly samples from the CA_0.50%_ group exhibited significantly greater acidity than those from the CA_0%_ and CA_0.75%_ groups (*p* < 0.05). Furthermore, the total phenolic acid content in the royal jelly samples from the experimental groups ranged from 18.426 mg/g to 40.735 mg/g. Specifically, the total phenolic acid content was slightly higher in the royal jelly from the CA_0.50%_ group than in that from the CA_0%_ group, whereas the total phenolic acid content was significantly greater in the royal jelly from the CA_0.75%_ group than in both the CA_0.50%_ and CA_0%_ groups (*p* < 0.05).

## 4. Discussion

This study suggested that adding CA to the diet significantly extends the lifespan of isolated reared worker bees, which aligns with the findings of previous studies and confirms the positive regulatory effect of CA on bee longevity [38,39]. Building upon earlier results and findings by Deodoro M Brighenti [38], who suggested that 0.3% CA could extend bee lifespan, the subsequent feeding experiments refined the CA proportions to 0.25%, 0.50%, 0.75%, and 1.00% for further analysis. The underlying mechanism may involve the role of CA in bee physiological metabolism and immune function. CA, a natural antioxidant, likely helps reduce oxidative stress levels in bees, slowing cell apoptosis and tissue damage and thereby prolonging lifespan [40,41]. Additionally, CA might influence bee lifespan by modulating their feeding behavior and energy metabolism. Existing studies indicate that CA stimulates appetite and promotes food intake and energy absorption, enabling bees to obtain more nutrients, thereby increasing their energy reserves and extending lifespan [42,43]. In this study, when the CA concentration in the diet reached 0.75%, the worker bees exhibited the longest survival time, suggesting that this concentration of CA had the best physiological regulatory and appetite-promoting effects. In practical beekeeping, an extended lifespan implies that bee colonies can maintain better physiological conditions and health during unfavorable periods, such as wintering, reducing the risk of death and ultimately improving the overall survival rate of the colonies, thereby aiding in healthy beekeeping practices. We also observed that adding 0.75% CA significantly promoted the consumption of feed by bees, indicating that CA might enhance the palatability or taste of feed, increasing the preference of bees for feed [41]. Furthermore, CA participates in various metabolic pathways within organisms, including the tricarboxylic acid cycle, fatty acid synthesis, amino acid metabolism, and sugar metabolism [44,45,46,47]. CA involvement in the tricarboxylic acid cycle could improve the digestion and absorption efficiency of pollen, allowing bees to more effectively utilize the nutrients present in the pollen [48,49]. However, the internal mechanisms through which CA promotes bee feed digestion require further research.

Under the isolated rearing conditions, we observed that the acinar surface area of the CA_0.75%_ group was greater than that of the CA_0.50%_ group, indicating that the addition of CA to feed promoted the growth and development of worker bee hypopharyngeal glands, prolonged the developmental peak, and thus extended the secretion time of royal jelly. This finding suggested that feeding with CA may stimulate the development of wax glands in worker bees. However, the underlying physiological or social mechanisms require further study. CA might influence the development of bee glands through pathways such as fatty acid synthesis and amino acid metabolism, thereby accelerating glandular development and secretion [44]. Ultimately, this may enhance cellular proliferation and differentiation in the glands, improving the quality of royal jelly [50,51]. Studies indicate a correlation between normal bee gland development and royal jelly production [52]. When bee glands are adequately developed and active, substances they secrete such as enzymes and hormones can more effectively stimulate the production of royal jelly. Our study revealed that adding CA to feed significantly affects various components of the produced royal jelly, with a 0.50% ratio indicating the most pronounced effect. CA supplementation in feed notably reduces the moisture content of royal jelly and increases its acidity, contributing to its antibacterial properties and preservation. Furthermore, adding 0.50% citric acid to feed significantly enhanced the 10-HDA content in royal jelly while slightly reducing the total phenolic acid content, indicating the role of CA in regulating the functional components of royal jelly.

## 5. Conclusions

This study analyzed the effects of CA on bee lifespan and gland development, confirming that adding 0.75% CA to feed effectively prolongs bee lifespan and promotes gland development. The addition of CA to feed helps enhance the quality of the produced royal jelly, particularly when CA is added at a 0.50% ratio, significantly increasing the content of 10-HDA in royal jelly. Considering both aspects, adding CA to feed at concentrations between 0.50% and 0.75% in actual beekeeping practice can effectively increase bee nutrition and royal jelly quality. This practice aids in healthy beekeeping and artificial feed development, contributing to the sustainable growth in apiculture.

## Figures and Tables

**Table 1:** Effect of the proportion of CA added to feed on the quality of the royal jelly.

Groups	Moisture Content/%	10-HDA/%	Total Protein/%	Water-Soluble Protein g/100 g	Total Sugar (as Glucose)/%	Acidity (1 mol/L NaOH)/(mL/100 g)	Total Phenolic Acid mg/g
CA_0%_	66.80 ± 0.20 ^a^	1.725 ± 0.005 ^a^	12.65 ± 0.25 ^ab^	9.309 ± 0.501 ^a^	10.28 ± 0.32 ^a^	32.25 ± 0.25 ^a^	20.949 ± 2.522 ^a^
CA_0.50%_	66.30 ± 0.10 ^b^	1.995 ± 0.005 ^b^	12.30 ± 0.30 ^a^	9.969 ± 0.347 ^a^	9.78 ± 0.08 ^b^	34.70 ± 0.10 ^b^	22.272 ± 2.522 ^a^
CA_0.75%_	63.45 ± 0.15 ^c^	1.755 ± 0.005 ^a^	13.10 ± 0.20 ^b^	9.672 ± 0.379 ^a^	10.31 ± 0.09 ^a^	31.65 ± 0.25 ^a^	37.728 ± 3.007 ^b^

Note: Different lowercase letters in the same column indicate significant differences (*p* < 0.05).

## Data Availability

Several data, which were generated during the current study, are not publicly available, but are available from the corresponding author on reasonable request.
